# Analysis of a Segmented Annular Coplanar Capacitive Tilt Sensor with Increased Sensitivity

**DOI:** 10.3390/s16010133

**Published:** 2016-01-21

**Authors:** Jiahao Guo, Pengcheng Hu, Jiubin Tan

**Affiliations:** Department of Automation Measurement and Control Engineering, Harbin Institute of Technology, D-403 Science Park, 2 Yikuang Street, Harbin 150080, China; gwojaho@gmail.com (J.G.); jbtan@outlook.com (J.T.)

**Keywords:** segmented annular coplanar capacitor, structure optimization, tilt sensing, increased sensitivity

## Abstract

An investigation of a segmented annular coplanar capacitor is presented. We focus on its theoretical model, and a mathematical expression of the capacitance value is derived by solving a Laplace equation with Hankel transform. The finite element method is employed to verify the analytical result. Different control parameters are discussed, and each contribution to the capacitance value of the capacitor is obtained. On this basis, we analyze and optimize the structure parameters of a segmented coplanar capacitive tilt sensor, and three models with different positions of the electrode gap are fabricated and tested. The experimental result shows that the model (whose electrode-gap position is 10 mm from the electrode center) realizes a high sensitivity: 0.129 pF/° with a non-linearity of <0.4% FS (full scale of ±40°). This finding offers plenty of opportunities for various measurement requirements in addition to achieving an optimized structure in practical design.

## 1. Introduction

Motivated by the fringing effect existing in conventional capacitors, different types of coplanar capacitive sensors have been proposed in recent years [1,2,3,4,5]. With the demand for lab-on-a-chip devices and the need for sensor miniaturization, coplanar capacitive sensors with interdigital electrodes [6,7,8,9,10] are proposed as one of the most used periodic electrodes configuration. Because of the unique structure in which the sensor electrodes lie in the same plane, specimens can be easily sensed or tested from one side of the sensor, instead of within the space between electrodes, which largely expands the application fields of capacitive sensors. By employing advanced manufacturing techniques, such coplanar electrodes can be fabricated very tightly, and a relatively high capacitance value can be easily and stably obtained compared with conventional methods. All these benefits make the coplanar capacitive sensor a popular option for applications in detecting food quality [1], water intrusion [2,3], relative humidity [4], and particulate matter [5].

In some particular situations, several works have been completed to design and characterize coplanar capacitive sensors to meet different measurement requirements [11] where the conventional rectangular electrodes are replaced with concentric annular ones [12,13]. Such annular coplanar capacitive sensors are superior in terms of rotational symmetry, and they also possess larger sensing zones. Accordingly, studies on their mathematical models have been conducted [14,15,16].

By setting the capacitor in cylindrical coordinates, Chen derived an electrostatic Green’s function from point charges using the Hankel transform method [14]. By dividing the electrodes into circular filaments and accumulating the respective charge distribution, the capacitance value was then calculated. Experiments demonstrated the capability for detecting water intrusion in radome structures. In [15], a simple closed-form solution for concentric coplanar capacitors was introduced by Cheng, where a Laplace equation on the electrical potential was solved by replacing a Dirichlet boundary condition with a Neumann one. A double-layered medium model of the capacitor was developed to simulate the stratum corneum and deep tissue layer of the body. Such concentric coplanar capacitors could be used for epidermal hydration sensing.

However, the research work mentioned above focused on an existing model of the annular capacitor, which consists of an inner central disk and an outer annulus. Further, their applications are limited within the material characteristics, instead of geometrical-dimension measurement. In the present paper, an analytical model of a segmented annular coplanar capacitor is proposed, which links the central angle (a geometrical dimension) to the capacitance value of a capacitor. We derive a mathematical expression of the capacitance by solving a Laplace equation with Hankel transform. A finite element model of the capacitor is built and solved to validate this analytical result. On the basis of the analysis result, the structure parameters of a tilt sensor with such segmented annular coplanar capacitors are optimized, and a corresponding sensitivity experiment demonstrates the feasibility and validity of the proposed analytical method.

**Figure 1:**
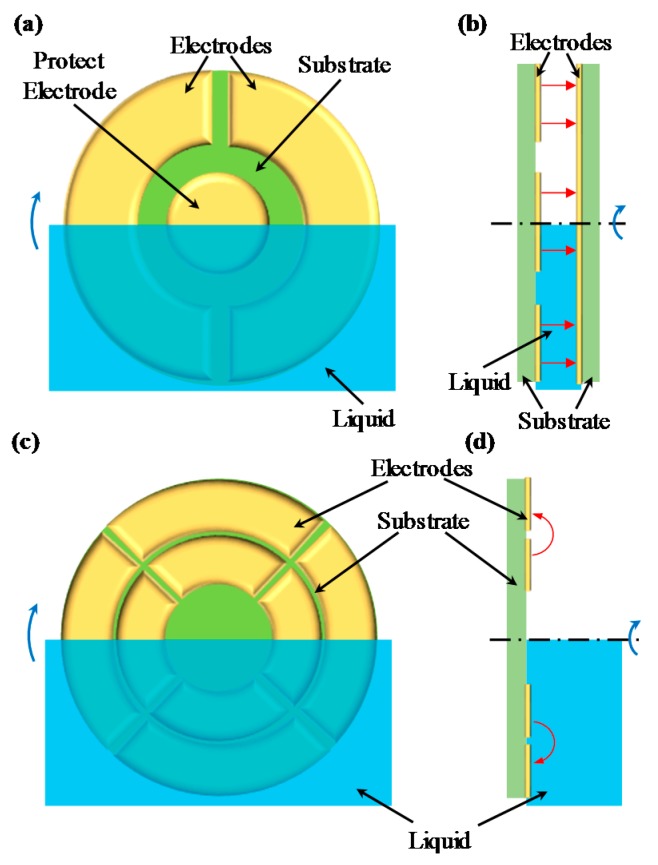
Schematic views of capacitive tilt sensors using parallel electrodes: (**a**) front view and (**b**) side view; schematic views of capacitive tilt sensors using annular coplanar electrodes: (**c**) front view and (**d**) side view.

Compared with conventional capacitive tilt sensors [17,18,19] using parallel electrodes shown in Figure 1a,b, which suffer from a large viscous drag lag and nonuniformity of the distance between two parallel electrodes, the proposed capacitive tilt sensors [20] using annular coplanar electrodes shown in Figure 1c,d are free from these problems and own an excellent performance.

## 2. Analytical Model

**Figure 2:**
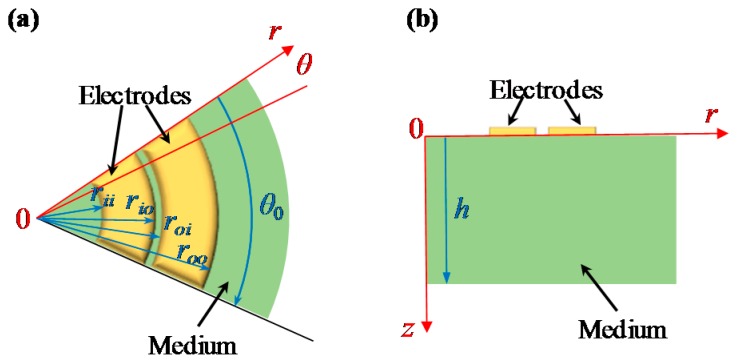
Schematic views of a segmented concentric annular coplanar capacitor: (**a**) top view; and (**b**) section view.

Figure 2 shows that a segmented annular coplanar capacitor consists of two concentric electrodes with central angle *θ*_0_. *r*_ii_ and *r*_io_ are inner and outer radii of the inner annular electrode, respectively, and *r*_oi_ and *r*_oo_ are inner and outer radii of the outer annular electrode, respectively. The coplanar capacitance consists of three parts: two fringing capacitances on two sides of electrodes and one normal capacitance between electrodes. In a coplanar capacitive sensor, we make use of the fringing effect to measure other physical quantities, rather than normal capacitance. Consequently, the electrode thickness should be very thin and neglectable compared with other dimensions [2]. Due to the symmetry, the electric-field distributions on two sides of electrodes are similar, and we concentrate on an analytical model of the coplanar capacitor with a medium on one side for convenience. Assume that the permittivity and thickness of the medium are *ε* and *h* separately. Potential *φ* in the medium satisfies the Laplace equation Δ*φ* = 0.

When the origin of a cylindrical-coordinate system is set at the center of the electrodes, the Laplace equation is expressed as

(1) $$\Delta \varphi =\frac{1}{r}\frac{\partial }{\partial r}\left(r\frac{\partial \varphi }{\partial r}\right)+\frac{1}{r^{2}}\frac{\partial^{2}\varphi }{\partial \theta^{2}}+\frac{\partial^{2}\varphi }{\partial z^{2}}=0$$

where *r* is the radial distance, *θ* is the azimuth, and *z* is the depth. Because the electrodes are swept along the peripheral direction, azimuth *θ* is not a determinant factor of electric potential *φ* in the medium, and Equation (1) could be rewritten as

(2) $$\Delta \varphi =\frac{\partial^{2}\varphi }{\partial r^{2}}+\frac{1}{r}\frac{\partial \varphi }{\partial r}+\frac{\partial^{2}\varphi }{\partial z^{2}}=0$$

The boundary conditions of the Laplace equation yield the following conclusions:

(A) At the interface between electrodes and medium, the potential difference between the inner and outer annular electrode is expressed as Δ*V*, *i.e.*,
  (3) $${\varphi |}_{z=0,r_{\mathrm{ii}}<r<r_{\mathrm{io}}}-{\varphi |}_{z=0,r_{\mathrm{oi}}<r<r_{\mathrm{oo}}}=\Delta V$$
(B) In other areas of this interface, the *z*-direction component of the electric field intensity is zero.
  (4) $${\frac{\partial \varphi }{\partial z}|}_{z=0,0<r<r_{\mathrm{ii}}}={\frac{\partial \varphi }{\partial z}|}_{z=0,r_{\mathrm{io}}<r<r_{\mathrm{oi}}}={\frac{\partial \varphi }{\partial z}|}_{z=0,r_{\mathrm{oo}}<r<r_{\infty }}=0$$
(C) At the bottom surface of the medium, the z-direction component of the electric field intensity is zero.
  (5) $${\frac{\partial \varphi }{\partial z}|}_{z=h}=0$$
(D) Through sector integration of the electric density, the approximate equations of the electric quantity on the inner and the outer annular electrodes can be expressed as
  (6) $$Q\approx \int_{0}^{\theta_{0}}\int_{r_{\mathrm{ii}}}^{r_{\mathrm{io}}}\varepsilon \left(-{\frac{\partial \varphi }{\partial z}|}_{z=0}\right)rdrd\theta \approx \varepsilon \frac{{r_{\mathrm{io}}}^{2}-{r_{\mathrm{ii}}}^{2}}{2}\theta_{0}\left({-\frac{\partial \varphi }{\partial z}|}_{z=0,r_{\mathrm{ii}}<r<r_{\mathrm{io}}}\right)$$
  (7) $$-Q\approx \int_{0}^{\theta_{0}}\int_{r_{\mathrm{oi}}}^{r_{\mathrm{oo}}}\varepsilon \left(-{\frac{\partial \varphi }{\partial z}|}_{z=0}\right)rdrd\theta \approx \varepsilon \frac{{r_{\mathrm{oo}}}^{2}-{r_{\mathrm{oi}}}^{2}}{2}\theta_{0}\left({-\frac{\partial \varphi }{\partial z}|}_{z=0,r_{\mathrm{oi}}<r<r_{\mathrm{oo}}}\right)$$

Subsequently, we obtain another two conditions, *i.e.*,

(8) $${-\frac{\partial \varphi }{\partial z}|}_{z=0,r_{\mathrm{ii}}<r<r_{\mathrm{io}}}\approx \frac{2Q}{\varepsilon \left({r_{\mathrm{io}}}^{2}-{r_{\mathrm{ii}}}^{2}\right)\theta_{0}}$$

(9) $${-\frac{\partial \varphi }{\partial z}|}_{z=0,r_{\mathrm{oi}}<r<r_{\mathrm{oo}}}\approx -\frac{2Q}{\varepsilon \left({r_{\mathrm{oo}}}^{2}-{r_{\mathrm{oi}}}^{2}\right)\theta_{0}}$$

To solve the Laplace equation, a zeroth-order Hankel transform is employed, and Equation (2) is expressed as

(10) $$\frac{\partial^{2}\Psi \left(\xi ,z\right)}{\partial z^{2}}-\xi^{2}\Psi \left(\xi ,z\right)=0$$

A general solution is easily obtained as

(11) $$\Psi \left(\xi ,z\right)=K_{1}e^{-\xi z}+K_{2}e^{\xi z}$$

For boundary condition C, we have

(12) $${\frac{\partial \Psi \left(\xi ,z\right)}{\partial z}|}_{z=h}=0$$

For boundary conditions B and D, we have

(13) $${-\frac{\partial \Psi \left(\xi ,z\right)}{\partial z}|}_{z=0}=\frac{2Q}{\varepsilon \theta_{0}}\cdot \left[\frac{r_{io}J_{1}\left(\xi r_{\mathrm{io}}\right)-r_{\mathrm{ii}}J_{1}\left(\xi r_{\mathrm{ii}}\right)}{\xi \left({r^{2}}_{\mathrm{io}}-{r^{2}}_{\mathrm{ii}}\right)}-\frac{r_{\mathrm{oo}}J_{1}\left(\xi r_{\mathrm{oo}}\right)-r_{\mathrm{oi}}J_{1}\left(\xi r_{\mathrm{oi}}\right)}{\xi \left({r^{2}}_{\mathrm{oo}}-{r^{2}}_{\mathrm{oi}}\right)}\right]$$

By solving Equation (11) using transformed boundary Equations (12) and (13), we obtain a particular solution for Equation (10).

(14) $$\Psi \left(\xi ,z\right)=\frac{2Q}{\varepsilon \theta_{0}}\frac{\cosh\left[\xi \left(h-z\right)\right]}{\xi \sinh(\xi h)}\cdot \left[\frac{r_{\mathrm{io}}J_{1}\left(\xi r_{\mathrm{io}}\right)-r_{\mathrm{ii}}J_{1}\left(\xi r_{\mathrm{ii}}\right)}{\xi \left({r^{2}}_{\mathrm{io}}-{r^{2}}_{\mathrm{ii}}\right)}-\frac{r_{\mathrm{oo}}J_{1}\left(\xi r_{\mathrm{oo}}\right)-r_{\mathrm{oi}}J_{1}\left(\xi r_{\mathrm{oi}}\right)}{\xi \left({r^{2}}_{\mathrm{oo}}-{r^{2}}_{\mathrm{oi}}\right)}\right]$$

Therefore, the inverse zeroth-order Hankel transform of Equation (14) helps solve the initial Laplace equation.

(15) $$\varphi \left(r,z\right)=\int_{0}^{\infty }\Psi \left(\xi ,z\right)J_{0}\left(\xi r\right)\xi d\xi$$

We utilize the average value over the surface integration as an approximation of the electrode electric potential, *i.e.*,

(16) $${\varphi |}_{z=0,r_{\mathrm{ii}}<r<r_{\mathrm{io}}}=\frac{\theta_{0}\int_{r_{\mathrm{ii}}}^{r_{\mathrm{io}}}{\varphi \left(r,z\right)|}_{z=0}rdr}{\frac{\theta_{0}}{2}\left({r^{2}}_{\mathrm{io}}-{r^{2}}_{\mathrm{ii}}\right)}$$

(17) $${\varphi |}_{z=0,r_{\mathrm{oi}}<r<r_{\mathrm{oo}}}=\frac{\theta_{0}\int_{r_{\mathrm{oi}}}^{r_{\mathrm{oo}}}{\varphi \left(r,z\right)|}_{z=0}rdr}{\frac{\theta_{0}}{2}\left({r^{2}}_{\mathrm{oo}}-{r^{2}}_{\mathrm{oi}}\right)}$$

By combining the boundary condition in A in Equation (3), we obtain

(18) $$\Delta V=\frac{4Q}{\varepsilon \theta_{0}}\int_{0}^{\infty }\frac{{\left[\frac{r_{\mathrm{io}}J_{1}\left(\xi r_{\mathrm{io}}\right)-r_{\mathrm{ii}}J_{1}\left(\xi r_{\mathrm{ii}}\right)}{\xi \left({r^{2}}_{\mathrm{io}}-{r^{2}}_{\mathrm{ii}}\right)}-\frac{r_{\mathrm{oo}}J_{1}\left(\xi r_{\mathrm{oo}}\right)-r_{\mathrm{oi}}J_{1}\left(\xi r_{\mathrm{oi}}\right)}{\xi \left({r^{2}}_{\mathrm{oo}}-{r^{2}}_{\mathrm{oi}}\right)}\right]}^{2}}{\tanh(\xi h)}d\xi$$

According to the definition of capacitance, an analytical expression of capacitance value *C* can be obtained as

(19) $$C=\frac{Q}{\Delta V}=\varepsilon \cdot \theta_{0}\cdot \frac{1}{4\int_{0}^{\infty }\frac{{\left[\frac{r_{\mathrm{io}}J_{1}\left(\xi r_{\mathrm{io}}\right)-r_{\mathrm{ii}}J_{1}\left(\xi r_{\mathrm{ii}}\right)}{\xi \left({r^{2}}_{\mathrm{io}}-{r^{2}}_{\mathrm{ii}}\right)}-\frac{r_{\mathrm{oo}}J_{1}\left(\xi r_{\mathrm{oo}}\right)-r_{\mathrm{oi}}J_{1}\left(\xi r_{\mathrm{oi}}\right)}{\xi \left({r^{2}}_{\mathrm{oo}}-{r^{2}}_{\mathrm{oi}}\right)}\right]}^{2}}{\tanh(\xi h)}d\xi }$$

**Figure 3:**
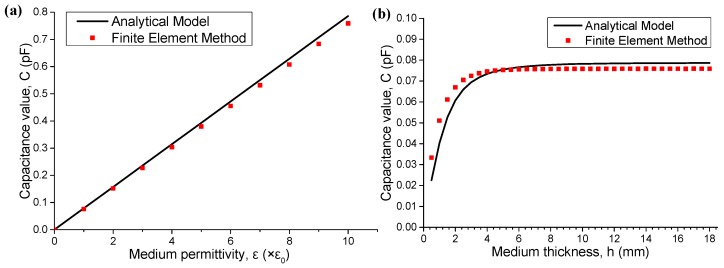
Capacitance values of the capacitors with (**a**) different medium permittivity *ε* and (**b**) different medium thickness *h*.

To validate the analytical model in Equation (19), capacitance value *C* is also calculated by a finite element method. The electrostatic field analysis based on a finite element method is available in ANSYS, a finite element program. Firstly, we concentrate on the parameters of the medium, permittivity *ε* and thickness *h*. Figure 3a shows that capacitance value *C* is strictly proportional to permittivity *ε*, where the result obtained from finite element method agrees well with that of the proposed analytical model. The default settings of other parameters are listed as follows: *h* = 10 mm, *θ*_0_ = 60°, *r*_ii_ = 7 mm, *r*_io_ = 9 mm, *r*_oi_ = 9.5 mm and *r*_oo_ = 11.5 mm. Figure 3b shows that capacitance value *C* increases with medium thickness *h* when medium permittivity *ε* is 1 × ε_0_. When *h* increases beyond a critical value of approximately 6 mm, the ascending trend disappears.

**Figure 4:**
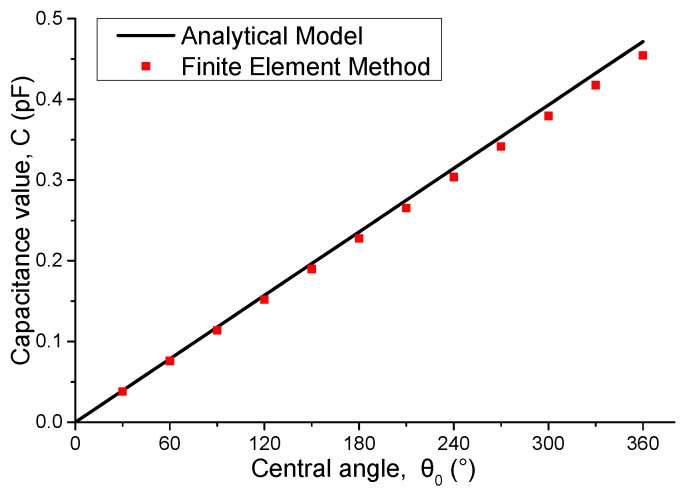
Capacitance values of the capacitors with different central angle *θ*_0_.

The next analysis is conducted under the condition that medium permittivity *ε* is fixed at 1 × ε_0_ and thickness is fixed at 15 mm, where ε_0_ is vacuum permittivity. We subsequently consider the geometric parameters of capacitor electrodes. Different values of central angle *θ*_0_ are set for electrodes, and both calculation and simulation results are shown in Figure 4. The capacitance value *C* is strictly proportional to central angle *θ*_0_ of the electrodes.

**Figure 5:**
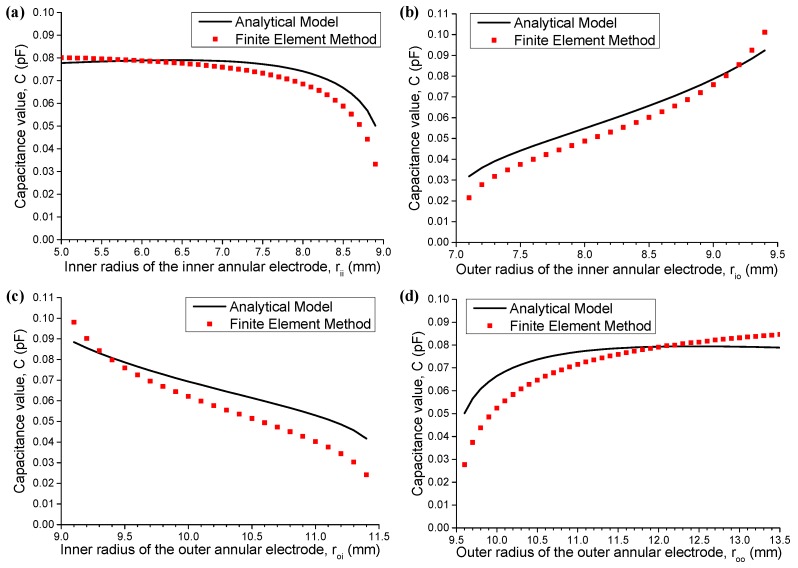
Capacitance values of the capacitors with different parameters in the radial direction: (**a**) *r*_io_ = 9 mm, *r*_oi_ = 9.5 mm, and *r*_oo_ = 11.5 mm; (**b**) *r*_ii_ = 7 mm, *r*_oi_ =9.5 mm, and *r*_oo_ = 11.5 mm; (**c**) *r*_ii_ = 7 mm, *r*_io_ = 9 mm, and *r*_oo_ = 11.5 mm; and (**d**) *r*_ii_ = 7 mm, *r*_io_ = 9 mm, and *r*_oi_ = 9.5 mm.

Finally, four radial parameters of capacitor electrodes, namely, *r*_ii_, *r*_io_, *r*_oi_, and *r*_oo_, are individually studied while central angle *θ*_0_ is fixed as a default setting of 60°. Figure 5 shows the results. In general, *C* increases with the geometric size of capacitor electrodes. When *r*_ii_ increases from 5.0 mm to 7.0 mm, as shown in Figure 5a, *C* remains almost unchanged. The same result can be easily observed in Figure 5d when *r*_oo_ is in the interval from 11.5 mm to 13.5 mm. This result indicates that for a fixed distance between two electrodes, the electrode size’s contribution to the increase in capacitance value *C* becomes increasingly lesser when radial width reaches a certain extent. For example, 2 mm is a radial width limit for electrodes in the proposed model when the distance between two electrodes is approximately 0.5 mm.

The overall trend of two curves shown in Figure 5 agrees well, which indicates that the proposed analytical model is a suitable approximation of a real capacitor. Let us consider a situation in which the distance between two electrodes remains the same, whereas the radial length of electrodes becomes smaller, namely, *r*_ii_ approaches *r*_io_ = 9 mm, as shown in Figure 5a, and r_oo_ approaches *r*_oi_ = 9.5 mm, as shown in Figure 5d. We notice that the difference between analytical model and result from the finite element method becomes larger. It is because the fringing effect between two electrodes in the real model is negligible at this time, and the proposed analytical model does not apply any more. Further, when the distance between two annular electrodes becomes larger than 1 mm, namely, *r*_io_ approaches *r*_ii_ = 7 mm (Figure 5b) and r_oi_ approaches *r*_oo_ = 11.5 mm (Figure 5c), the proposed analytical model is not sufficiently accurate. It is because the normal capacitance is dominant in the capacitance value *C* in this condition.

Table 1 shows a concise conclusion on how different parameters affect capacitance value *C*. Based on the analysis result mentioned above, we optimize the structure parameters of a tilt sensor that uses segmented annular coplanar capacitors shown in Figure 1c,d.

**Table 1:** Contributions of different control parameters to capacitance value *C*.

Control Parameters	Contribution to *C*
Central angle *θ*_0_	Strictly proportional to *C*; designed to satisfy various needs
Medium thickness *h*	Supposed to be large enough to guarantee a large *C*
Inner radius of inner annular electrode *r*_ii_	Not necessarily too small to guarantee a large *C*; designed according to the value of *r*_io_
Outer radius of inner annular electrode *r*_io_	Determining the distance between two electrodes and assumed to be small enough to guarantee a large *C*
Inner radius of outer annular electrode *r*_oi_	
Outer radius of outer annular electrode *r*_oo_	Not necessarily too large to guarantee a large *C*; designed according to the value of *r*_oi_

## 3. Implementation and Experiment

**Figure 6:**
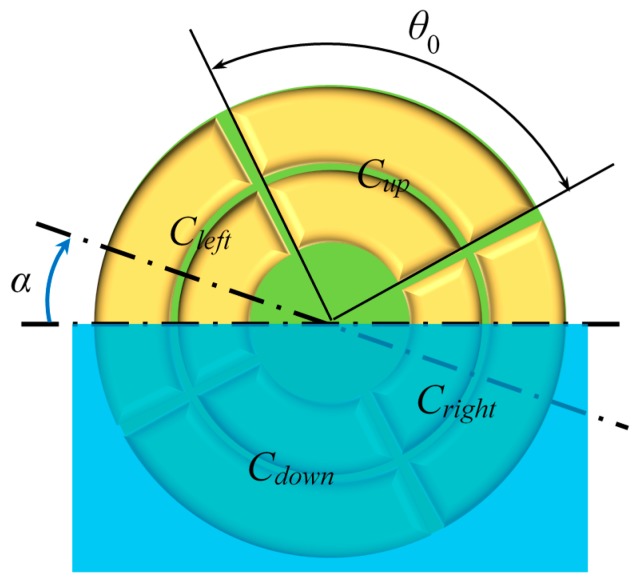
Schematic view of a tilt sensor with four segmented concentric annular coplanar capacitors.

According to the analytical result, capacitance value *C* of a segmented annular coplanar capacitor is linearly proportional to medium permittivity ε as well as central angle *θ*_0_. From this fact, a tilt sensor is designed, and its sensing mechanism is shown in Figure 6.

Four segmented annular coplanar capacitors with central angle *θ*_0_ = 88° mentioned above are centrally symmetrically distributed on a dielectric substrate, as shown in Figure 6. These four capacitors are half-immersed in non-conducting liquid of which the level crosses the common center of the electrodes. Each segmented capacitor consists of two parts: the capacitance in the substrate side and the capacitance at the opposite side. We designate the thicknesses of the substrate and the liquid as *h*_sub_ and *h*_liq_, respectively.

The tilt sensor rotates clockwise (or anti-clockwise) with a small inclination angle *α*, whereas the liquid remains relatively static. Consequently, the capacitance values of the left and right capacitors change with the rotation. Four capacitance values in the tilt sensor can be described by the following equations:

(20) $$C_{\mathrm{down}}=K_{\mathrm{sub}}\cdot \frac{22\pi }{45}\cdot \varepsilon_{\mathrm{sub}}+K_{\mathrm{liq}}\cdot \frac{22\pi }{45}\cdot \varepsilon_{\mathrm{liq}}$$

(21) $$C_{\mathrm{left}}=K_{\mathrm{sub}}\cdot \frac{22\pi }{45}\cdot \varepsilon_{\mathrm{sub}}+K_{\mathrm{liq}}\cdot \left(\frac{11\pi }{45}+\alpha \right)\cdot \varepsilon_{\mathrm{air}}+K_{\mathrm{liq}}\cdot \left(\frac{11\pi }{45}-\alpha \right)\cdot \varepsilon_{\mathrm{liq}}$$

(22) $$C_{\mathrm{up}}=K_{\mathrm{sub}}\cdot \frac{22\pi }{45}\cdot \varepsilon_{\mathrm{sub}}+K_{\mathrm{liq}}\cdot \frac{22\pi }{45}\cdot \varepsilon_{\mathrm{air}}$$

(23) $$C_{\mathrm{right}}=K_{\mathrm{sub}}\cdot \frac{22\pi }{45}\cdot \varepsilon_{\mathrm{sub}}+K_{\mathrm{liq}}\cdot \left(\frac{11\pi }{45}-\alpha \right)\cdot \varepsilon_{\mathrm{air}}+K_{\mathrm{liq}}\cdot \left(\frac{11\pi }{45}+\alpha \right)\cdot \varepsilon_{\mathrm{liq}}$$

where the ratio parameters *K*_sub_ and *K*_liq_ satisfy the following equations:

(24) $$K_{\mathrm{sub}}=\frac{1}{4\int_{0}^{\infty }\frac{{\left[\frac{r_{\mathrm{io}}J_{1}\left(\xi r_{\mathrm{io}}\right)-r_{\mathrm{ii}}J_{1}\left(\xi r_{\mathrm{ii}}\right)}{\xi \left({r^{2}}_{\mathrm{io}}-{r^{2}}_{\mathrm{ii}}\right)}-\frac{r_{\mathrm{oo}}J_{1}\left(\xi r_{\mathrm{oo}}\right)-r_{\mathrm{oo}}J_{1}\left(\xi r_{\mathrm{oi}}\right)}{\xi \left({r^{2}}_{\mathrm{oo}}-{r^{2}}_{\mathrm{oi}}\right)}\right]}^{2}}{\tanh(\xi h_{\mathrm{sub}})}d\xi }$$

(25) $$K_{\mathrm{liq}}=\frac{1}{4\int_{0}^{\infty }\frac{{\left[\frac{r_{\mathrm{io}}J_{1}\left(\xi r_{\mathrm{io}}\right)-r_{\mathrm{ii}}J_{1}\left(\xi r_{\mathrm{ii}}\right)}{\xi \left({r^{2}}_{\mathrm{io}}-{r^{2}}_{\mathrm{ii}}\right)}-\frac{r_{\mathrm{oo}}J_{1}\left(\xi r_{\mathrm{oo}}\right)-r_{\mathrm{oo}}J_{1}\left(\xi r_{\mathrm{oi}}\right)}{\xi \left({r^{2}}_{\mathrm{oo}}-{r^{2}}_{\mathrm{oi}}\right)}\right]}^{2}}{\tanh(\xi h_{\mathrm{liq}})}d\xi }$$

Through simple derivation from Equations (20)–(24), we obtain the equation for *α*.

(26) $$\alpha =\frac{11\pi }{45}\cdot \frac{C_{\mathrm{right}}-C_{\mathrm{left}}}{C_{\mathrm{down}}-C_{\mathrm{up}}}$$

We should note that *C*_up_ and *C*_down_ are constant when the tilt angle is within ±44° because the corresponding electrodes are fully exposed in air or immersed in liquid. Further, under this condition, the sensitivity of this tilt sensor can be analyzed as expressed in the following equation:

(27) $$C_{\mathrm{right}}-C_{\mathrm{left}}=2\cdot K_{\mathrm{liq}}\cdot \left(\varepsilon_{\mathrm{liq}}-\varepsilon_{\mathrm{air}}\right)\cdot \alpha =C_{\mathrm{dif}}\cdot \alpha$$

We introduce parameter *C*_dif_ to simplify the analysis process. In Equation (27), *C*_dif_ represents the capacitance value of a segmented annular coplanar capacitor whose parameters are listed as follows: *θ*_0_ = 2 rad, *ε* = *ε*_liq_−*ε*_air_, and *h* = *h*_liq_. Radial sizes *r*_ii_, *r*_io_, *r*_oi_, and *r*_oo_ are the same as those of the capacitors in this study. Theoretically speaking, a larger *C*_dif_ results in better sensitivity of the proposed tilt sensor.

According to the analysis presented in Section 2, a smaller distance between two electrodes can easily lead to a large *C*_dif_. Because of the limit in the conventional technology, in most cases, radial distance *D* = *r*_oi_−*r*_io_, cannot be fabricated at a small-scale level. Here, we utilize the printed-circuit-board technology to fabricate the sensor, where a 0.2-mm-wide distance can be accurately ensured. Under this condition, the inner and outer boundaries of electrodes are set as *r*_ii_ = 7 mm and *r*_oo_ = 11.5 mm after an overall consideration because 4.5 mm is sufficiently wide to maintain a large capacitance value. In reality, the permittivity of air *ε*_air_ is constant, and the non-conducting liquid with a larger permittivity *ε*_liq_ is preferred where glycerol (*ε*_liq_ ≈ 42.5 × ε_0_ ) is employed. By considering liquid thickness *h*_liq_, 15 mm is sufficient.

**Figure 7:**
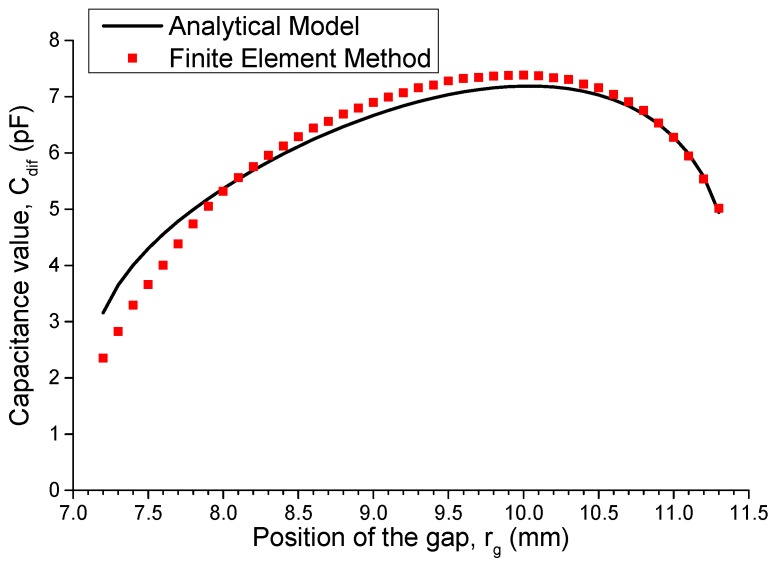
Capacitance values with different gap positions (*r*_ii_ = 7 mm, *r*_oo_ = 11.5 mm, *θ*_0_ = 2 rad, *h* = 15 mm, and *ε* = 41.5 × ε_0_).

Under these conditions, we optimize the values of r_io_ and r_oi_, namely, the position of the gap *r*_g_ = (*r*_io_ + *r*_oi_)/2, using the proposed analytical model as well as the finite element method. Figure 7 shows that when the gap moves from the inner to the outer boundary, *C*_dif_ first increases and then decreases, reaching a peak of 7.382 pF when *r*_g_ is equal to 10 mm. Finally, we choose *r*_io_ = 9.9 mm and *r*_oi_ = 10.1 mm to realize the best sensitivity of the tilt sensor.

**Figure 8:**
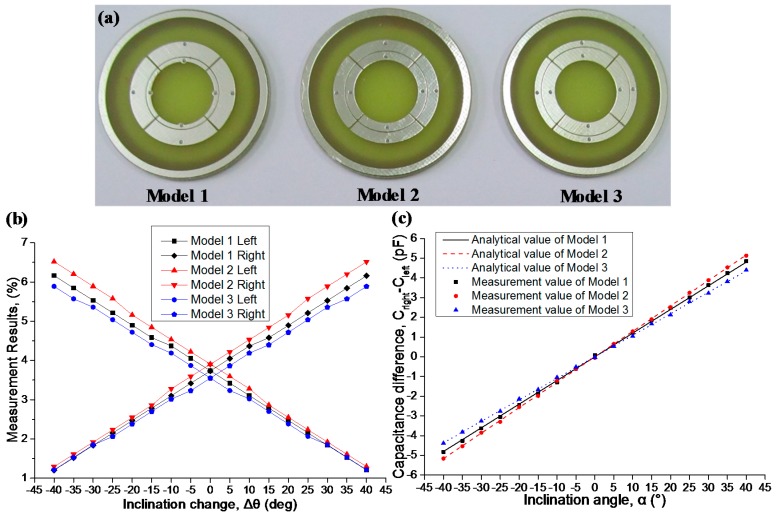
(**a**) three models with different structure parameters; (**b**) percentage change of *C*_right_ and *C*_left_ respect to the inclination change in three models; (**c**) corresponding sensitivity comparison results.

To verify the accuracy of the analysis process, three models with different gap position (*r*_g_ is equal to 9 mm, 10 mm, and 11 mm for Models 1, 2, and 3, respectively) are fabricated utilizing printed circuit board (PCB) technology, as shown in Figure 8a. The electrodes of the planar capacitors are made of metal copper covered with the tin which prevent the oxidation process of metal copper. The dielectric substrate is made of fiberglass resin, which is a commonly used material for PCB. Limited by the conventional technology, the electrode thickness is about 0.035 mm. Eight holes on each model are utilized for precise measurement of the capacitance value. Measurement tests on the relationship between *C*_right_ − *C*_left_ and *α* are conducted on a standard tilt platform, and Figure 8b shows the percentage change of *C*_right_ and *C*_left_ with respect to the inclination change in three models. In Figure 8c, *C*_right_ − *C*_left_ and *α* accurately dovetail with a linear relationship derived from Equation (27). Model 2 yields the highest sensitivity of 0.129 pF/°, followed by Model 1 (0.120 pF/°) and Model 3 (0.109 pF/°). Corresponding non-linearity of three models is calculated and found to be below 0.5% FS (full scale of ±40°) for Models 1 and 3 and below 0.4% FS for Model 2.

**Figure 9:**
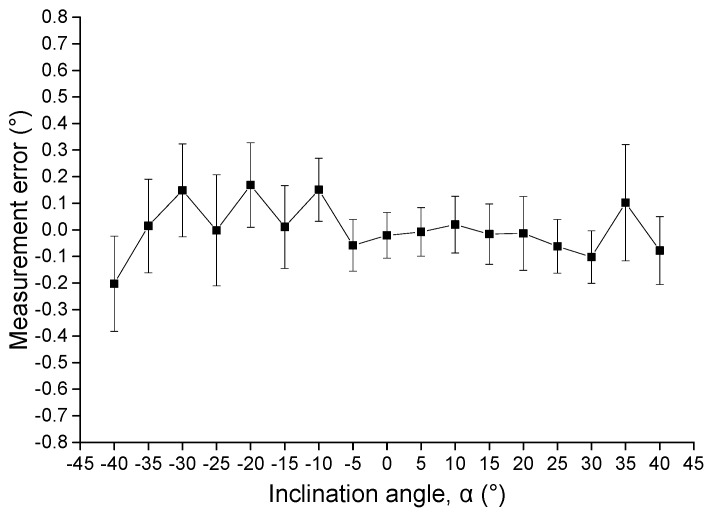
Accuracy test of the proposed tilt sensor with Model 2.

In addition, the accuracy test of Model 2 is also performed on the standard tilt platform. When the inclination angle of the platform varies by 5° per step from −40° to 40°, the capacitance values of four segmented coplanar capacitors are recorded, and corresponding α is calculated using Equation (26). The results for 10 measurement times are shown in Figure 9, which indicates that a 0.4° accuracy is achieved. It should be mentioned that the highest measurement errors are found for negative inclination angles. One possible reason might be the manufacturing non-uniformity in electrode gap. When Model 2 rotates anti-clockwise (with negative inclination angles), the left electrodes get into liquid gradually and the increase of *C*_left_ is not strictly proportional to the inclination change. The non-uniformity in the left electrode gap might cause a higher measurement error for negative inclination angles.

We chose 88° as the central angle of fabricated devices because of a tradeoff between the accuracy and the measurement range of the proposed tilt sensor. The central angle of 88° means that the segmenting angle between two adjacent inner annular electrodes is 2°. In terms of a central angle larger than 88°, adjacent annular electrodes are too close to each other that the cross capacitance between two coplanar capacitors might influence the accuracy of the final measurement result. In terms of a central angle smaller than 88°, the segmenting angle, as well as the insensitive zone of the tilt sensor, becomes larger. Then, the measurement range decreases as a result. However, such a design is not unique and other parameters for coplanar capacitors could be tried in future work.

## 4. Conclusions

An investigation on a segmented annular coplanar capacitor has been presented, and its analytical model has been established. By solving a Laplace equation with Hankel transform, a mathematical expression of the capacitance value is derived. The finite element method verifies the analytical result well. The dimension parameters of the coplanar capacitor are individually studied, and we obtain a general principle on their contributions to the capacitance value. Consequently, we analyze and optimize the structure parameters of a segmented coplanar capacitive tilt sensor utilizing the proposed analytical model. Three models with different positions of the electrode gap are fabricated and tested. The experiment results show that Model 2 (*r*_g_ = 10 mm) yields a high sensitivity: 0.129 pF/° with a non-linearity of <0.4% FS and an accuracy of 0.4° is achieved. When the total width for two electrodes is fixed, the width of the inner annular electrode should be larger than the width of the outer annular electrode to realize the best solution. The optimal width ratio *K*_wid_ is related with both inner radii of inner annular electrode *r*_ii_ and outer radii of outer annular electrode *r*_oo_. This finding offers plenty of opportunities for various measurement requirements in addition to achieving an optimized structure in practical design.
